# Role of neutrophil extracellular trap and immune infiltration in atherosclerotic plaque instability: Novel insight from bioinformatics analysis and machine learning

**DOI:** 10.1097/MD.0000000000034918

**Published:** 2023-09-22

**Authors:** Tingting Hu, Xiaomin Chen

**Affiliations:** a Health Science Center, Ningbo University, Ningbo, China; b Department of Cardiology, The First Affiliated Hospital of Ningbo University, Ningbo, China.

**Keywords:** atherosclerosis, immune infiltration, machine learning, neutrophil extracellular trap, unstable atherosclerotic plaque

## Abstract

The instability of atherosclerotic plaques increases the risk of acute coronary syndrome. Neutrophil extracellular traps (NETs), mesh-like complexes consisting of extracellular DNA adorned with various protein substances, have been recently discovered to play an essential role in atherosclerotic plaque formation and development. This study aimed to investigate novel diagnostic biomarkers that can identify unstable plaques for early distinction and prevention of plaque erosion or disruption. Differential expression analysis was used to identify the differentially expressed NET-related genes, and Gene Ontology and Kyoto Encyclopedia of Genes and Genomes analyses were performed. We filtered the characteristic genes using machine learning and estimated diagnostic efficacy using receiver operating characteristic curves. Immune infiltration was detected using single-sample gene set enrichment analysis and the biological signaling pathways involved in characteristic genes utilizing gene set enrichment analysis were explored. Finally, miRNAs- and transcription factors-target genes networks were established. We identified 8 differentially expressed NET-related genes primarily involved in immune-related pathways. Four were identified as capable of distinguishing unstable plaques. More immune cells infiltrated unstable plaques than stable plaques, and these cells were predominantly positively related to characteristic genes. These 4 diagnostic genes are involved in immune responses and the modulation of smooth muscle contractility. Several miRNAs and transcription factors were predicted as upstream regulatory factors, providing further information on the identification and prevention of atherosclerotic plaques rupture. We identified several promising NET-related genes (AQP9, C5AR1, FPR3, and SIGLEC9) and immune cell subsets that may identify unstable atherosclerotic plaques at an early stage and prevent various complications of plaque disruption.

## 1. Introduction

Cardiovascular disease (CVD) is a leading cause of death globally.^[1]^ Atherosclerosis (AS), the underlying pathogenesis of CVD, is a chronic progressive inflammatory disease, characterized by the initiation and development of fibrofatty lesions within the subendothelial intimate layer of the vessel wall.^[2,3]^ Although atheromatous plaques form gradually and remain clinically silent for a long time, they can cause death when the plaque ruptures.^[4]^ The stability of atherosclerotic plaques is the principal reason for acute cardiovascular and cerebrovascular adverse events and increases the risk of acute myocardial infarction and ischemic stroke.^[5]^ Unstable plaques, also called vulnerable plaques, are characterized by a large necrotic lipid core, thin fibrous cap, plaque angiogenesis, intraplaque hemorrhage, and microcalcification accompanied by an inflammatory response.^[6]^ These histological changes make vulnerable plaques more susceptible to rupture, leading to local thrombosis or embolism, thereby causing fatal complications. Hence, early identification of vulnerable plaques is essential to preventing erosion or disruption.^[7]^

One of the primary variables contributing to AS growth and plaque rupture is immune infiltration.^[8,9]^ Immune mechanisms coordinate all phases of the atherosclerotic plaque life cycle. The most crucial immune cells involved in the occurrence and development of AS are mononuclear macrophages that can phagocytize normal or modified lipoproteins. Furthermore, they stimulate inflammatory responses by releasing a variety of proinflammatory mediators and matrix metalloproteinases (MMPs), which ultimately result in cell death. As macrophages die, lipids and tissue components are released, creating a prothrombotic necrosis lipid kernel that serves as one of the building blocks of destabilized plaques.^[10]^ Helper T (Th) and regulatory T cells are 2 cell subtypes that differ from naive CD4^+^ T cells and are involved in AS immune responses.^[11]^ Regulatory T cells act as an anti-atherosclerotic counterbalance to Th1 cells’ pro-atherosclerotic effects.^[12,13]^ By modulating immune responses through the secretion of antibodies, cytokines, and antigens, B lymphocytes function in both local and systemic immune responses to affect the development of atherosclerotic plaques.^[14]^ However, the specific characteristics of immune cell dysregulation associated with plaque instability remain unclear.

Active neutrophils discharge neutrophil extracellular traps (NETs), mesh-like complexes consisting of extracellular DNA containing various protein mediators such as histones and neutrophil granule proteins.^[15]^ Owing to NETs’ capacity to capture and eliminate various infections, they were once believed to be neutrophils’ natural defensive mechanism.^[16,17]^ However, progress in research has led to the discovery of NETs that are also associated with the pathophysiological mechanisms of many disorders.^[18]^ In recent years, several studies have focused on the crucial role that NETs play in the creation and development of atheromatous plaques and in blood vessel thrombosis.^[19]^ NETs are the primary drivers of the self-amplifying loop of inflammation and cell death in AS.^[20,21]^ Endothelial and tissue impairment, NET generation, and their interactions work together to exacerbate plaque destabilization.^[22–24]^ Additionally, NET-related genes (NRGs) have been linked to atherosclerotic plaques. Neutrophil peptidylarginine deiminase 4 (PAD4) has a significant role in NET generation during the development of atherosclerotic plaque, as shown by research.^[25]^ In addition to inhibiting the generation of mouse and human NETs, PAD4 inhibition or knockout reduces atheromatous plaque lesions and postpones arterial thrombosis.^[26–29]^ However, a thorough and complete analysis of the role of NRGs in plaque destabilization is currently lacking.

Overall, this study aimed to examine the critical roles of diagnostic genes and infiltrating immune cells in the initiation and progression of atherosclerotic plaque destabilization.

## 2. Materials and Methods

### 2.1. Data acquisition and processing

Gene expression profiles of patients with AS were downloaded from the GSE41571 and GSE120521 datasets of the gene expression omnibus database (https://www.ncbi.nlm.nih.gov/geo/). The Affymetrix Human Genome U133 Plus 2.0 Array identified 5 instances of unstable atherosclerotic plaques and 6 examples of stable plaques in the GSE41571 dataset.^[30]^ Four stabilized and 4 destabilized atheromatous plaque samples were included in the GSE120521 dataset, which was generated using GPL16791 (Illumina HiSeq 2500 Homo sapiens).^[31]^ The raw microarray expression matrix was then standardized using the normalized quantile function of the preprocessCore package in the R software. Using the platform’s annotation information, the probe expression matrix was transformed into a gene expression matrix. The average value was derived to identify gene expression when numerous probes matched an identical gene.

**Figure 1.**
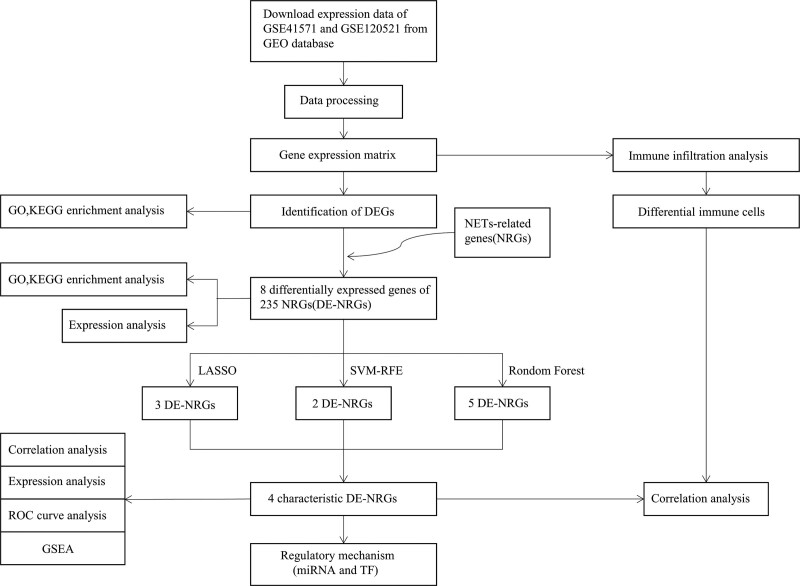
Flowchart of the present study.

Using the Combat function in the sva package, the expression profiles of the GSE41571 and GSE120521 datasets were mixed, and batch effects were directly eliminated.^[32]^ Principal component analysis was employed to display the data using the R packages FactoMineR and Factoextra to estimate the group effects in the collected dataset. Informed consent and ethical approval were not required because all study data were obtained from the gene expression omnibus database. Figure 1 illustrates the bioinformatics analysis used in our study.

### 2.2. Selection of NRGs

Sixty-nine NRGs were retrieved from previous studies^[33–35]^ and additional 190 NRGs were extracted from the Kyoto encyclopedia of genes and genomes (KEGG) pathway database (https://www.kegg.jp/pathway/hsa04613). Finally, the integration of the 2 gene sets indicated that 235 NRGs were produced earlier. Supplemental Table 1, Supplemental Digital Content, http://links.lww.com/MD/J621 provides detailed gene information.

### 2.3. Differential expression analysis of NRGs

Differential expression analysis was performed on unstable and stable atheromatous plaque samples via the limma R package to confirm differentially expressed genes (DEGs).^[36]^ The significance threshold |log fold change| >0.5 and adjust *P* value < .05 were regarded as the screening criteria. The R tools ggplot2 and pheatmap were used to create heatmaps and volcano plots to illustrate the findings. Finally, we identified the intersection between DEGs and NRGs as differentially expressed NRGs (DE-NRGs).

### 2.4. Functional enrichment analysis

Relevant data regarding the roles of genes and their products were supplied in an organized and calculable manner using the Gene Ontology (GO) database.^[37]^ The KEGG is a well-known resource for detailed investigation of genomes, biological pathways, diseases, and pharmaceuticals.^[38]^ GO annotation and KEGG pathway analyses were performed using the “ClusterProfiler” R package to evaluate probable biological functions and underlying mechanisms of unstable and stable atherosclerotic plaques.^[39]^ Cellular components, biological processes, and molecular functions comprised the 3 primary GO terms used. Using the false discovery rate for multiple hypothesis testing and the Benjamini–Hochberg approach, the *P* value was corrected, and enrichment was only considered statistically significant when the adjusted *P* value was <.05. Based on the DEGs and DE-NRGs, GO and KEGG analyses were performed twice in this study.

### 2.5. Identification of characteristic genes

To identify the characteristic genes, 3 machine-learning approaches were adopted: random forest (RF), support vector machine-recursive feature elimination (SVM-RFE),^[40]^ and least absolute shrinkage and selection operator (LASSO) logistic regression. LASSO linear regression adjusts variable selection and regularization while fitting the generalized linear model. It is superior to simple linear regression when dealing with high-dimensional data. The glmnet R package was implemented to establish a LASSO regression predictive model.^[41]^ RF, an ensemble learning algorithm, is a classifier containing multiple decision trees that rank DE-NRGs according to the Gini importance measurement using the randomForest R package.^[42]^ Recursive feature elimination strategies, such as the SVM-RFE algorithm, outperformed the mean squared error and discriminant analysis using linear models. It was implemented to choose pertinent qualities and eliminate duplicate attributes using weighted vectors generated from the SVM.^[43]^ By selecting genes that appeared in at least two of the aforementioned algorithms, we identified 4 DE-NRGs as characteristic genes.

### 2.6. Receiver operating characteristic (ROC) curve evaluation and expression analysis

We used ROC curves to evaluate each characteristic gene’s diagnostic effectiveness in the integrated GSE41571 and GSE120521 datasets and calculated the area under the ROC curve to assign a value to it using the pROC function in the R package (v4.0.2). The diagnostic process was considered more successful when the area under curve (AUC) was close to 1. The last-resort diagnostic marker genes were characterized using an AUC > 0.7.^[44]^ The circlize R package was used to further explore the relationships among confirmed genes. Eight DE-NRG expression levels were compared between samples of unstable and stable atherosclerotic plaque using box plots produced by the “ggplot2” R package.

### 2.7. Immune cell infiltration analysis

We investigated immune cell subset variations between samples of unstable and stable plaques to better understand the immune cell features of atherosclerotic plaque instability. The single sample gene set enrichment analysis (ssGSEA), a GSEA extension approach, was implemented to measure the infiltrating levels of 23 types of immune cells among different atheromatous plaque specimens using the “gene set variation analysis” package.^[45]^ Immune cell infiltration was analyzed using the Wilcoxon rank-sum test, and box plots were drawn to compare the infiltrating fractions of individual immune cells among distinct plaque specimens using the ggplot R package. A correlation heatmap was constructed to depict the findings of Spearman correlation analysis, which was performed to reveal the association between the diverse invading immune cell subtypes.

### 2.8. Correlation analysis between diagnostic genes and infiltrating immune cells

The association between diagnostic genes and infiltrating immune cells was examined using immune infiltration analysis with ssGSEA algorithm and Spearman’s rank correlation test.^[46]^ The lollipop plots served to illustrate the relationships between characteristic genes and infiltrating immune cells.

### 2.9. Gene set enrichment analysis

To explore the potential biological functions and mechanisms of the diagnostic genes, based on the clusterProfiler package, we conducted GSEA. GSEA is a computerized approach used to evaluate alterations in biological pathways and activities in expression datasets.^[47]^ The Reactome Database was used to compile specified reference gene sets that are part of biological pathways.^[48]^ For the GSEA, *P* < .05 was considered statistically significant.

### 2.10. Construction of NET-related microribonucleic acids (miRNAs)- and transcription factors (TFs)-target gene networks

The RegNetwork (https://regnetworkweb.org/) online database was used to predict possible miRNAs and upstream TFs targeting diagnostic genes.^[49]^ NRGs-associated miRNA- and TF-target gene regulatory networks were constructed. The DE-NRGs derived from the merged datasets GSE41571 and GSE120521 were used to establish miRNAs- and TFs- DE-NRGs regulatory networks. The Cytoscape software (v3.8.2) was used to display the findings.

### 2.11. Statistically analysis

The R application (v4.0.2) was primarily used to deal with the data and perform statistical analysis. The Wilcoxon test was used to examine differences in expression between the 2 groups of atherosclerotic plaques. Spearman’s correlation analyses was used to assess whether there was a link between the 2 variables. The difference was deemed statistically significant at *P* < .05 for all two-sided statistical p-values.

## 3. Results

### 3.1. Identification of DEGs

**Figure 2.**
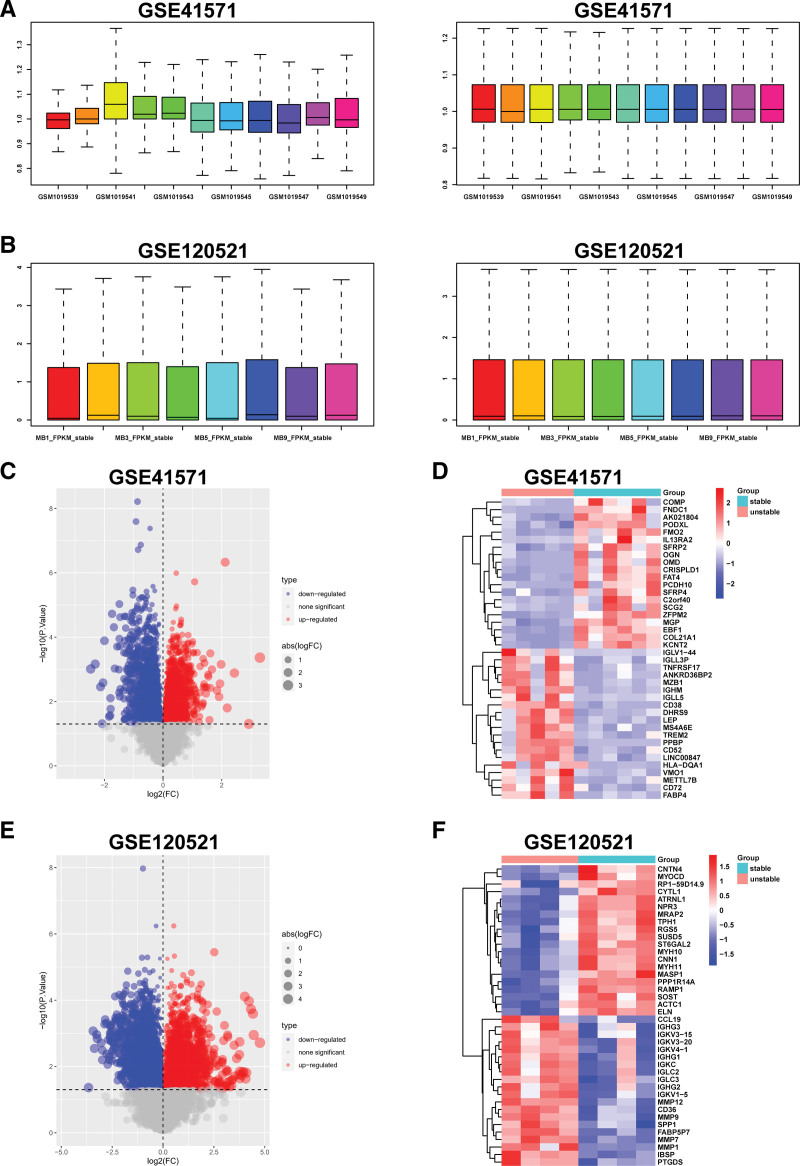
Identification of DEGs between unstable and stable atherosclerotic plaques. (A and B) Data preprocessing of GSE41571 and GSE120521 datasets. The left box diagram shows the sample distribution of each dataset before normalizing. The right box diagram shows the sample distribution of each dataset after normalizing. (C and D) Volcano plot and heatmap for DEGs identified from GSE41571 dataset. Red and blue represent DEGs with upregulated and downregulated gene expression, respectively. Each row displays DEGs, and each column refers to one of the samples of unstable or stable atherosclerotic plaque. (F and F) Volcano plot and heatmap for the DEGs identified from the GSE120521 dataset. DEGs = differentially expressed genes.

After accessing the GSE41571 and GSE120521 datasets, the raw gene expression matrix was normalized for subsequent analyses. In Figure 2A and B, the box plots display the sample distribution before and after the normalization of each dataset. To identify vulnerable atherosclerotic plaque-related genes, differential expression analysis was conducted. We obtained 891 DEGs between unstable and stable plaque specimens in the GSE41571 dataset, with 375 upregulated and 516 downregulated genes (Fig. 2C and D; Supplemental Table 2, Supplemental Digital Content, http://links.lww.com/MD/J622). In addition, we identified 3412 DEGs in the GSE120521 dataset, of which 1787 genes were upregulated in destabilized plaques compared to stabilized plaques, whereas 1625 genes were downregulated (Fig. 2E and F; Supplemental Table 3, Supplemental Digital Content, http://links.lww.com/MD/J623). Vascular smooth muscle contractility-related genes (CNN1, ACTC1, MYH10, and MYH11) showed lower expression levels in unstable plaques, whereas genes linked to antigen presentation (HLA-DQA1 and IG superfamily-related genes), inflammatory processes (CCL19, MMPs, and TNFRSF17), and lipid metabolism (FABP4, FABP5P7, and CD36) exhibited significantly higher expression levels. Our findings demonstrate that lipid accumulation, decreased vascular smooth muscle contraction, and immune responses contribute significantly to plaque instability.

### 3.2. Functional enrichment analysis and mechanism exploration

**Figure 3.**
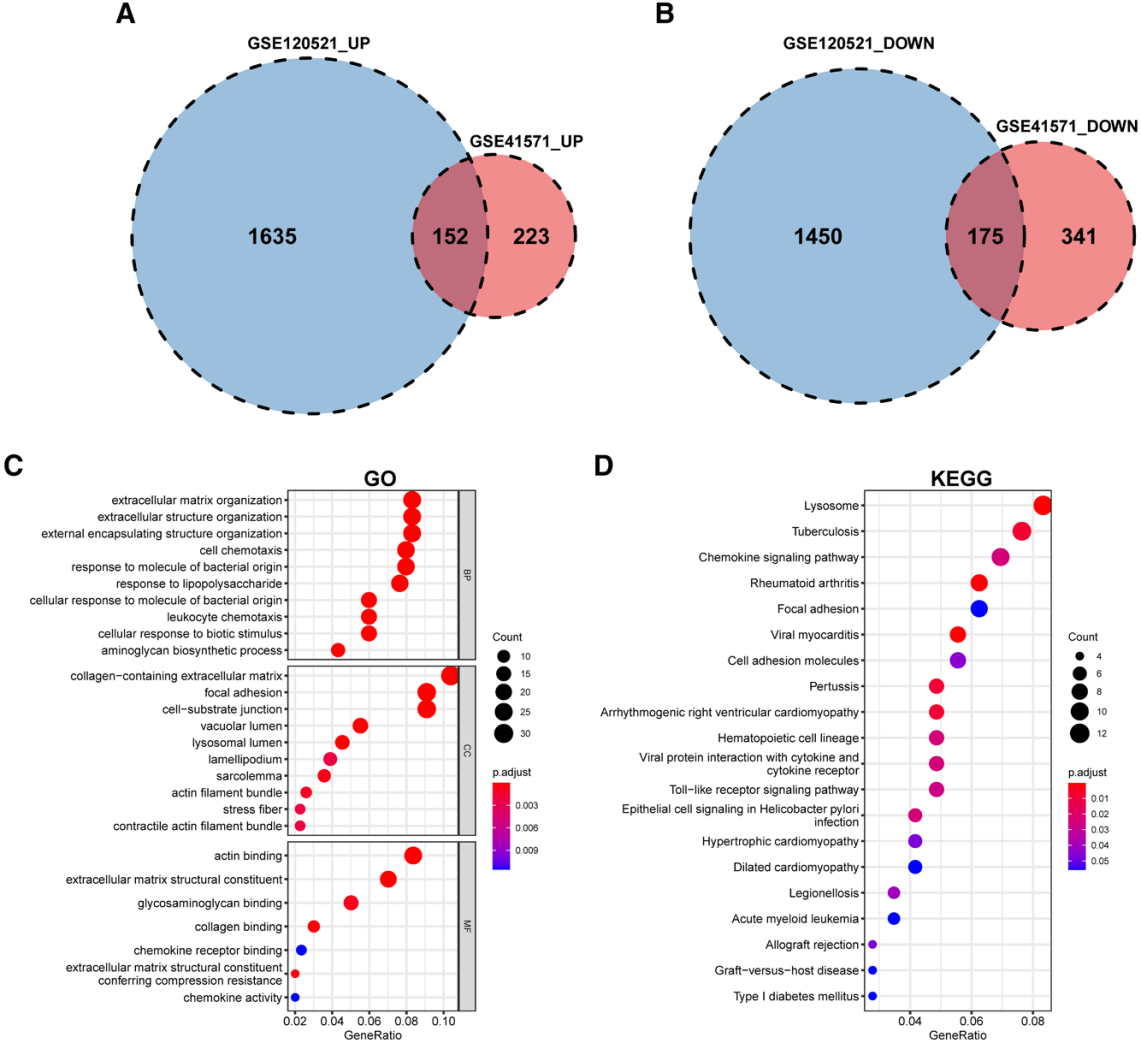
Enrichment analysis of DEGs. (A) Venn diagram shows that 152 upregulated genes are determined from the intersection of upregulated genes in GSE41571 and GSE120521 datasets. (B) Venn diagram shows that 175 downregulated genes are determined from the intersection of downregulated genes in GSE41571 and GSE120521 datasets. (C) GO analysis of the DEGs, including BPs, CCs, and MFs, respectively. The *y* axis represents different GO terms, the x-axis represents gene ratio enriched in relative GO terms, the circle size refers to gene numbers, and the color reveals the adjusted *P* value. (D) KEGG pathway analysis of the DEGs visualized by bubble plot. BPs = biological processes, CCs = cellular components, DEGs = differentially expressed genes, GO = Gene Ontology, MFs = molecular functions.

We overlapped the upregulated DEGs in the GSE41571 dataset with those in the GSE120521 dataset and obtained 152 overlapping upregulated DEGs (Fig. 3A; Supplemental Table 4, Supplemental Digital Content, http://links.lww.com/MD/J624). By comparing the downregulated DEGs in the GSE41571 dataset with those in the GSE120521 dataset, 175 downregulated DEGs were identified (Fig. 3B; Supplemental Table 4, Supplemental Digital Content, http://links.lww.com/MD/J624). Overall, 327 DEGs were identified between unstable and stable atherosclerotic plaque samples (Supplemental Table 4, Supplemental Digital Content, http://links.lww.com/MD/J624). Consistent with the GO analysis, DEGs were largely abundant in cell chemotaxis, collagen-containing extracellular matrix, cell substrate, contractile actin filament bundle, collagen binding, and actin binding (Fig. 3C). KEGG pathway analysis revealed that the DEGs were mostly involved in toll-like receptor signal transduction, focal adhesion, cell adhesion, and chemokine signaling pathways (Fig. 3D). These findings demonstrate that the primary contributing factors to plaque vulnerability are the immunological response, extracellular matrix, and cellular adhesion molecules.

### 3.3. Determination of DE-NRGs and functional enrichment analysis

**Figure 4.**
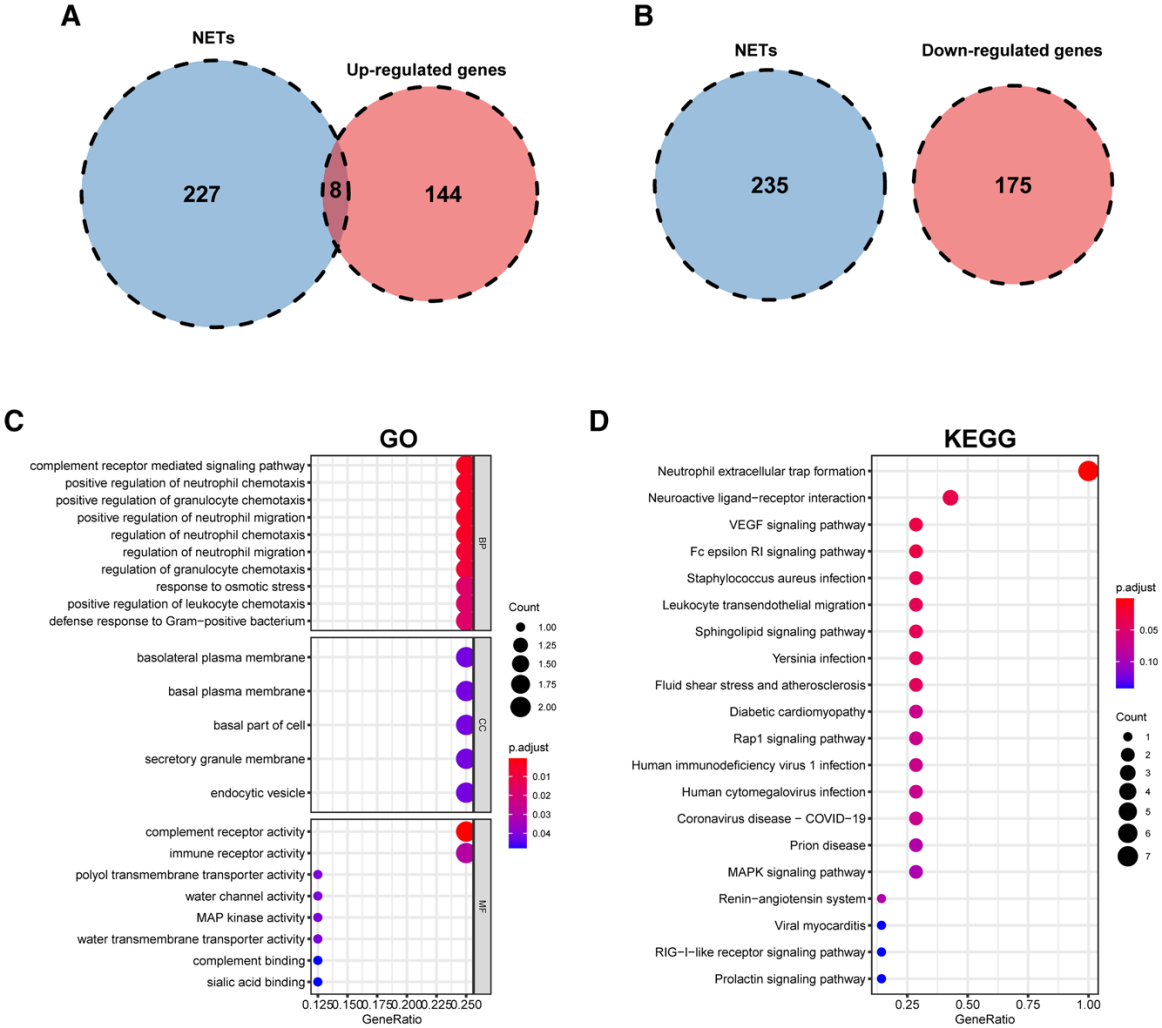
Determination of DE-NRGs and enrichment analysis. (A) Venn diagram of NRGs and upregulated DEGs. (B) Venn diagram of NRGs and downregulated DEGs. (C) GO analysis (BPs, CCs, and MFs) of DE-NRGs. (D) KEGG pathways enrichment analysis of DE-NRGs. BPs = biological processes, CCs = cellular components, DEGs = differentially expressed genes, GO = Gene Ontology, MFs = molecular functions, NRGs = NET-related genes.

**Figure 5.**
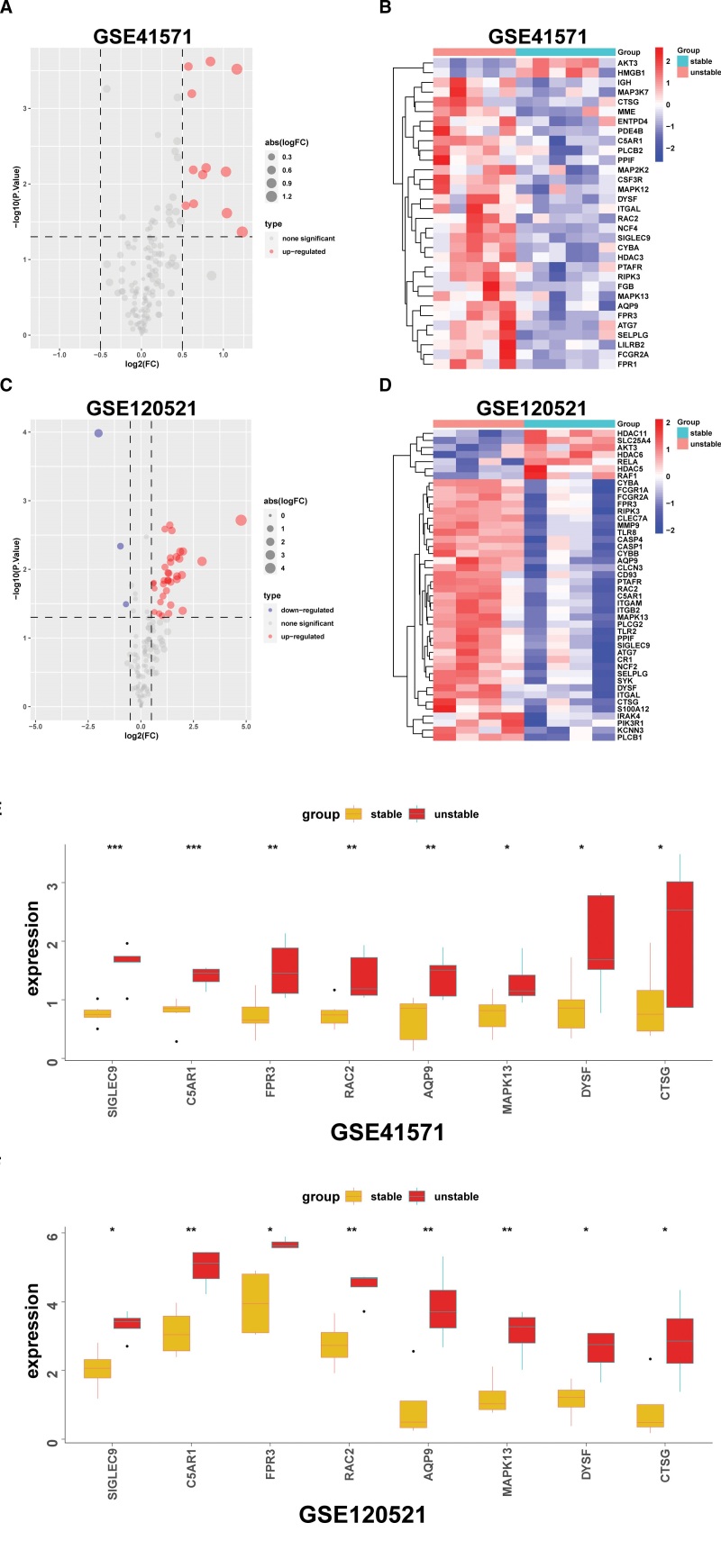
Expression levels of DE-NRGs in atherosclerotic plaques. (A and B) Volcano plot and heatmap of the DE-NRGs identified from the GSE41571 dataset. (C and D) Volcano plot and heatmap of the DE-NRGs identified from the GSE120521 dataset. (E and F) Overall expression histogram of DE-NRGs in atherosclerotic plaques in GSE41571 (upper) and GSE120521 (lower) datasets. Red for unstable plaque specimens, yellow for stable plaque specimens, and the horizontal axis indicates gene, and vertical axis indicates gene expression levels. *P < .05; **P < .01; ***P < .001. DE-NRGs = differentially expressed neutrophil extracellular traps related genes.

By integrating the 235 NRGs, 152 upregulated and 175 downregulated DEGs were identified, and 8 intersecting DE-NRGs, including SIGLEC9, C5AR1, FPR3, RAC2, AQP9, MAPK13, DYSF, and CTSG, were eventually identified (Fig. 4A and B; Supplemental Table 5, Supplemental Digital Content, http://links.lww.com/MD/J625). Volcano plots and heatmaps of DE-NRGs are displayed in Figure 5A–D. DE-NRGs showed significantly different expression patterns between unstable and stable atherosclerotic plaques in the GSE41571 and GSE20521 datasets. From the samples shown, the unstable plaque showed a considerably higher level of DE-NRG expression than the stable plaque (Fig. 5E and F), indicating their potential roles in plaque destabilization.

We conducted functional enrichment analyses of DE-NRGs to elucidate their functions and associated pathways in plaque instability. The GO annotation analysis revealed enrichment of the complement receptor-mediated signaling pathway, neutrophil chemotaxis, neutrophil migration, leukocyte chemotaxis, and granulocyte chemotaxis in biological processes. Moreover, the cellular components were enriched in the secretory granule membranes and endocytic vesicles. Furthermore, DE-NRGs were related to molecular functions such as complement receptor activity, immune receptor activity, complement binding, and sialic acid binding (Fig. 4C). According to KEGG pathway analysis, DE-NRGs participated in the following terms: neutrophil extracellular trap formation, vascular endothelial growth factor signaling pathways, Fc epsilon RI signal transduction pathways, and leukocyte transendothelial migration (Fig. 4D). These findings suggest that NET formation, neovascularization, immune responses, and inflammatory cell aggregation played essential roles in plaque instability.

### 3.4. Selection of characteristic genes via machine learning algorithms

**Figure 6.**
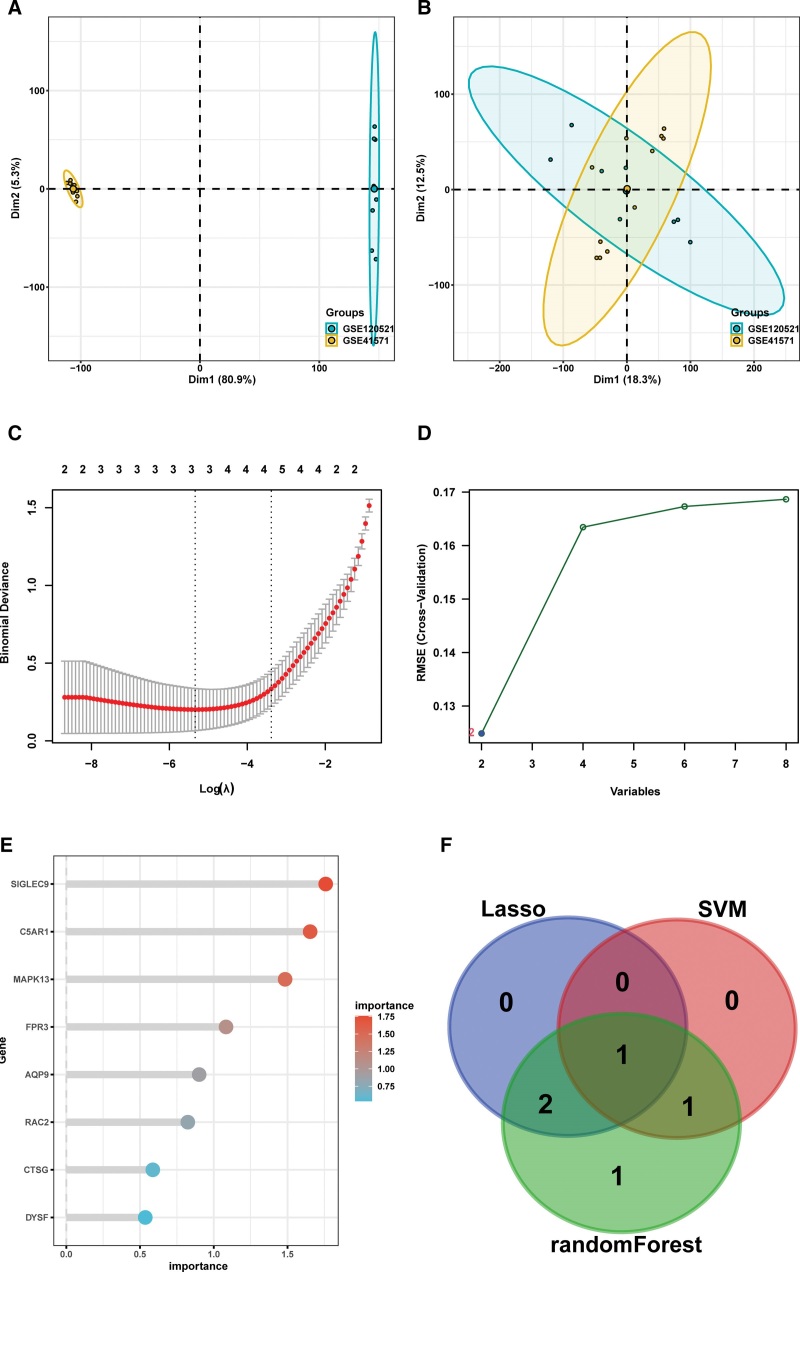
Selection of characteristic genes. (A) PCA plot shows the combined expression profiling of GSE41571 and GSE120521 cohorts before batch correction. (B) PCA plot of the integrated datasets after batch correction. (C) Screening of characteristic genes from DE-NRGs using the LASSO algorithm. (D) Feature NRGs were selected with the SVM-REF algorithm at the optimal point. (E) RF algorithm was conducted to rank DE-NRGs based on their relative importance. The abscissa represents mean decrease Gini, and the ordinate indicates DE-NRGs. (F) Venn diagram shows 4 characteristic genes shared by at least 2 algorithms above mentioned. DE-NRGs = differentially expressed NRGs, LASSO = least absolute shrinkage and selection operator, NRGs = neutrophil extracellular traps related genes, PCA = principal component analysis, RF = random forest, SVM-REF = support vector machine-recursive feature elimination.

We combined the GSE41571 and GSE120521 cohorts because of the limited sample size of each dataset and eliminated batch effects for the follow-up analysis (Fig. 6A and B). Eight DE-NRGs were used to filter the characteristic genes for the construction of a diagnostic model using 3 machine learning techniques. Among the 8 DE-NRGs, 3 candidate genes were identified using LASSO logistic regression (Fig. 6C). When the characteristic count for the SVM-RFE method was two, the classifier comprising C5AR1 and SIGLEC9 had the lowest false-positive rate (Fig. 6D). The RF algorithm ranked DE-NRGs according to the Gini coefficient (Fig. 6E). The intersection of 3 candidate genes from LASSO, 2 candidate genes screened by SVM-RFE, and the top 5 genes in terms of importance from RF were visualized using a Venn diagram (Fig. 6F). We selected genes that appeared in at least two of the algorithms mentioned above, and 4 DE-NRGs were confirmed as characteristic genes, including C5AR1, FPR3, AQP9, and SIGLEC9.

### 3.5. Diagnostic efficacy of characteristic genes in distinguishing unstable and stable atherosclerotic plaques

**Figure 7.**
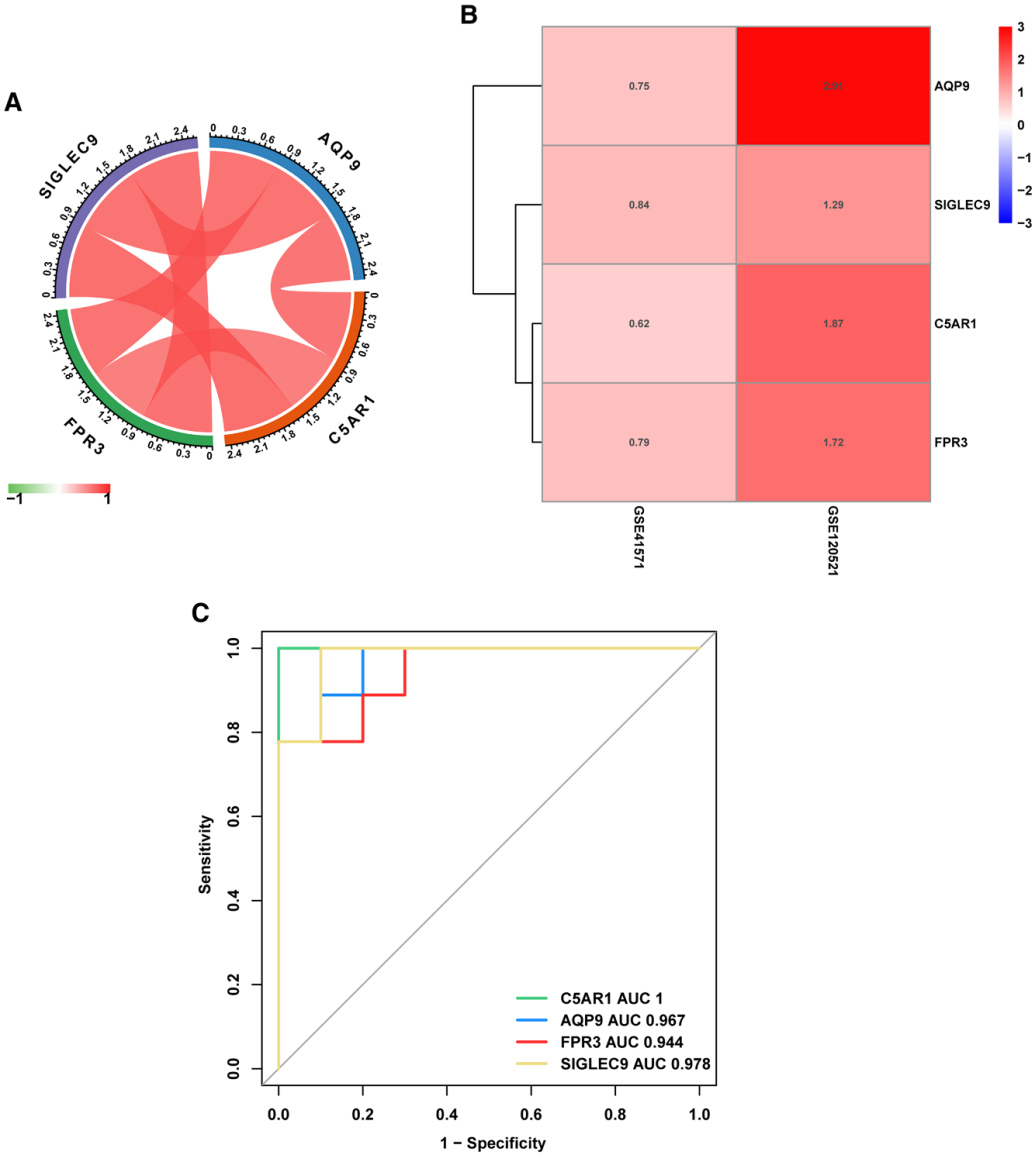
Diagnostic efficacy of characteristic genes in the prediction of atherosclerotic plaque instability. (A) Interactions between 4 characteristic genes at the molecular level. The red line represents a positive correlation and the green line represents a negative correlation. (B) Heatmaps depict the different expressions of characteristic genes in the GSE41571 and GSE120521 datasets, respectively. (C) The ROC curve estimates the diagnostic performance of characteristic genes in predicting atherosclerotic plaque instability. ROC = receiver operating characteristic.

We investigated the expression of the 4 characteristic genes in the GSE41571 and GES120521 datasets and found that these genes exhibited higher expression levels in unstable atherosclerotic plaque specimens (Fig. 7B). A correlation analysis of characteristic genes was also performed. The results showed remarkably positive interactions between these genes (Fig. 7A; Supplemental Figure 1, Supplemental Digital Content, http://links.lww.com/MD/J626). The integrated dataset was subjected to ROC curve analysis to assess the efficacy of characteristic genes as potential diagnostic biomarkers of plaque instability (Fig. 7C). Four characteristic genes (AUC > 0.7) were considered efficient diagnostic indicators. The AUC for C5AR1, which was 1, was the highest among the AUCs of the 4 characteristic genes. AUCs for SIGLEC9, AQP9, and FPR3 were 0.978, 0.967, and 0.944, respectively. Our findings showed that these 4 characteristic genes are highly effective in diagnosing atherosclerotic plaque instability.

### 3.6. Immune infiltrating cells analysis and its relationship with characteristic genes

**Figure 8.**
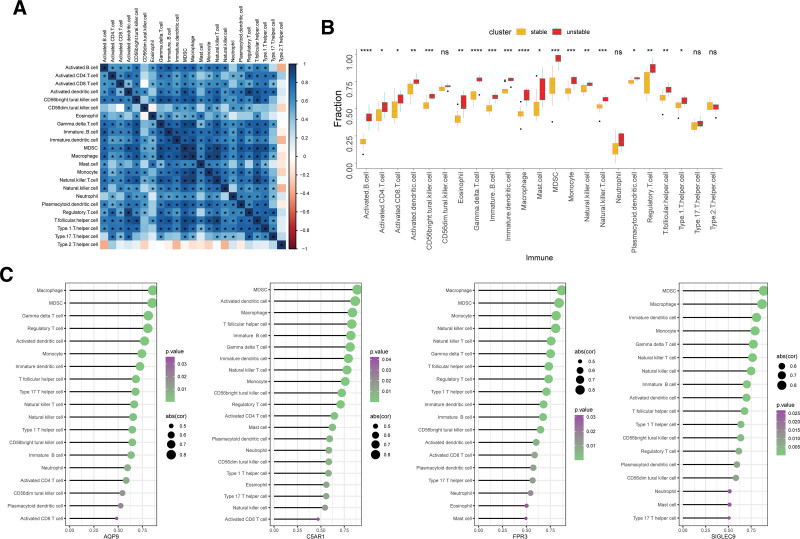
Immune cells infiltration analysis. (A) Heatmaps depict the correlations of 23 immune cells type compositions. Both horizontal and vertical axis demonstrates distinct immune cell subtypes. Blue indicates a positive correlation, and red indicates a negative correlation. * represents the significance of the correlation. (B) The comparison regarding the infiltrating levels of 23 kinds of immune cells between unstable and stable atherosclerotic plaque samples was visualized by histogram. (C) Correlation analysis of 4 characteristic genes with immune infiltrating cells, respectively. ^ns^*P* ≥ .05; *P < .05; **P < .01; ***P < .001; ****P < .0001.

Using enrichment analysis, we discovered that DE-NRGs were involved in pathways connected to the immune system. The ssGSEA algorithm was used to estimate the connection between NETs and immune cell infiltration characteristics in atherosclerotic plaque vulnerability, and it produced encouraging findings. Among the 23 types of immune cells, most innate and adaptive immune cells such as macrophages, activated B cells, monocytes, and immune B cells exhibited higher infiltration levels in unstable plaques than in stable plaques (Fig. 8B). In addition, there was a prominent correlation between the different immune-infiltrating cell subsets (Fig. 8A). According to the aforementioned studies, unstable atheromatous plaques elicited a stronger immune reaction.

We conducted a correlation evaluation to assess whether these characteristic genes were associated with immune infiltration to further investigate the functions of the diagnostic genes in immune responses. The investigation revealed that 4 characteristic genes, including AQP9, FPR3, C5AR1, and SIGLEC9, had a favorable connection with the infiltration of the majority of immune cells. For example, macrophages, myeloid-derived suppressor cells, monocytes, and immune B cells (Fig. 8C). Hence, these genes may promote plaque rupture by modulating immune cell infiltration.

### 3.7. Signaling pathways involved in characteristic genes

**Figure 9.**
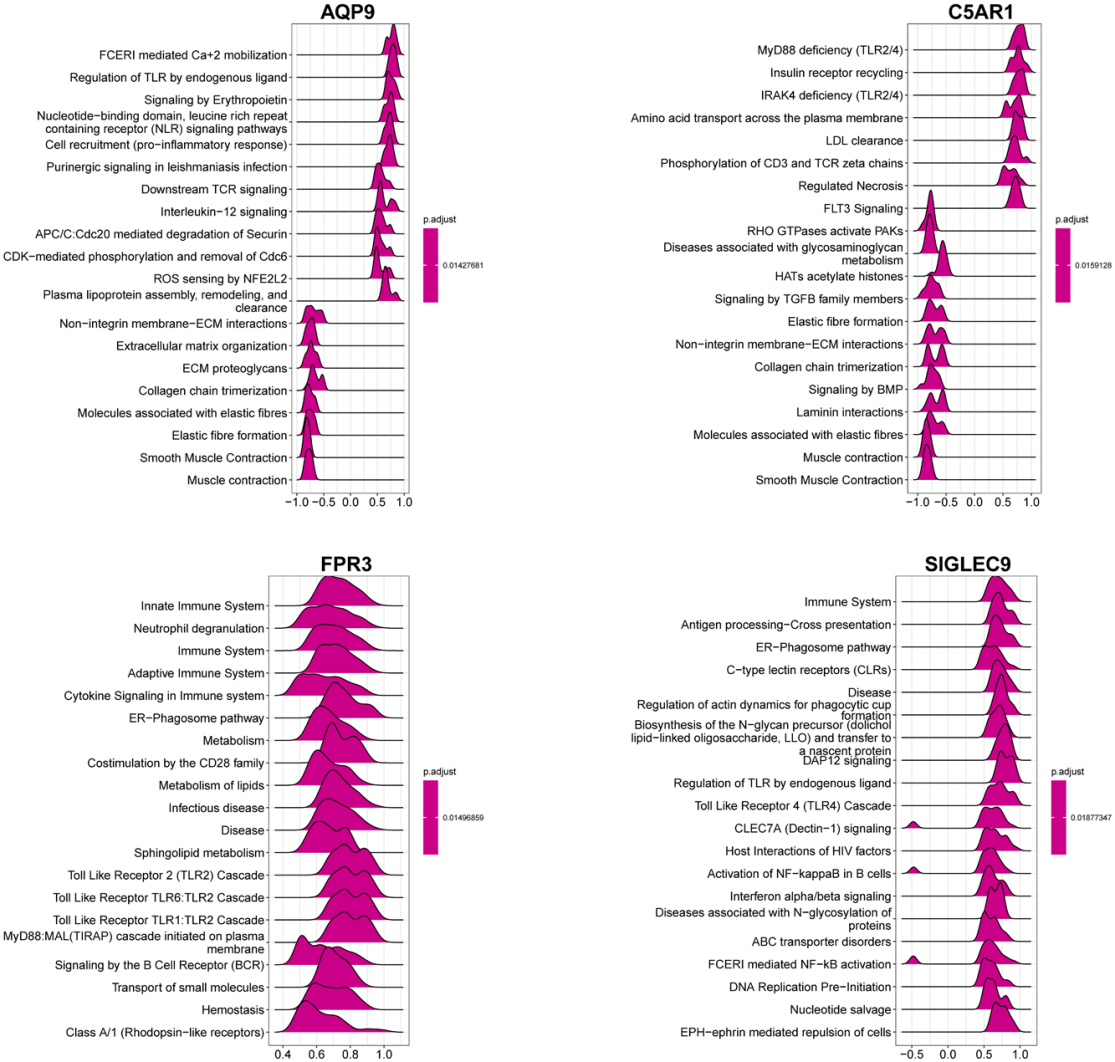
GSEA identifies signaling pathways involved in the characteristic genes. The top 20 signaling pathways that are significantly enriched in the high expression of characteristic genes. GSEA = gene set enrichment analysis.

Using GSEA, we investigated the signal transduction pathways related to the characteristic genes. The findings showed that all diagnostic genes, including AQP9, C5AR1, FPR3, and SIGLEC9, were associated with a favorable immunological response (such as nucleotide-binding domain, leucine-rich repeat-containing receptor signaling pathway, Toll-like receptor signaling pathway, B-cell receptor signaling pathway, and antigen processing-cross presentation) and were negatively linked to smooth muscle contraction and extracellular matrix organization (Fig. 9).

### 3.8. Prediction of miRNAs- and TF- target genes regulatory networks

**Figure 10.**
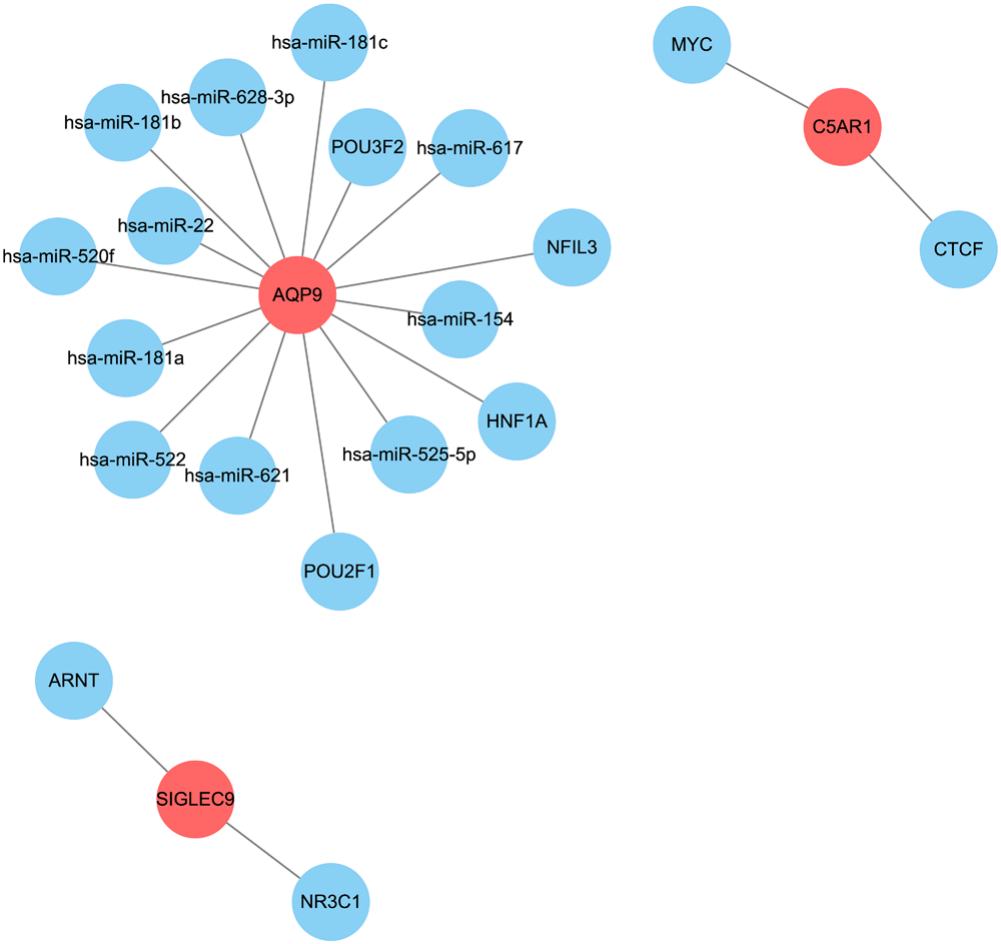
Prediction of miRNAs- and TFs-characteristic genes regulatory networks. The red dots represent characteristic genes, while the blue dots represent miRNAs or TFs. TFs = transcription factors.

The RegNetwork online database was accessed to predict potential miRNAs and transcription factors targeting diagnostic genes. The miRNA- and TF-characteristic genes regulatory networks are illustrated in Figure 10. The interaction networks comprised 3 characteristic genes, except for FPR3, for which no miRNAs or TFs were predicted. The AQP gene was regulated by multiple miRNAs and TFs, whereas the C5AR1 gene was only regulated by 2 TFs containing MYC and CTCF. In addition, TFs comprising ARNT and NR3C1 were predicted to regulate SIGLEC9 gene. The research on miRNA or TF regulatory networks may provide new perspectives for early disease detection and therapy, given that both miRNAs and TFs play substantial roles in the control of gene expression and disease development.

## 4. Discussion

In recent years, the principal cause of global mortality has been ischemic cardiovascular disorders, including acute coronary syndrome (ACS).^[50]^ The fibrous covering of vulnerable plaques is frequently disrupted, causing thrombus formation, which subsequently leads to ACS.^[51]^ Thus, it is necessary to identify and treat individuals with unstable plaques as soon as possible if they are at risk of ACS. Currently, several invasive and noninvasive imaging technologies such as intravenous ultrasound, optical coherence tomography, and angiography are used to assist in the identification of unstable plaques.^[52,53]^ However, these methods encounter several challenges. AS causes NETs generation, and these NETs accelerate the progression of AS and plaque formation, resulting in increased production of NETs. This triggers a favorable feedback cycle that promotes inflammation, which in turn increases the plaque burden and reduces the stability of atherosclerotic plaques.^[54,55]^

Vascular inflammation, a feature of AS, enhances the destabilization of atherosclerotic plaques.^[56]^ White blood cells and inflammation are now better understood as having an impact on the development of AS and its associated consequences in addition to conventional risk factors. T-cells,^[57]^ B-cells,^[14]^ dendritic cells,^[58]^ neutrophils,^[59]^ monocytes, and macrophages^[60]^ are only a few of the several pathogeneses that have been linked to this condition. Although the specific immunological mechanisms underlying plaque destabilization have been studied for decades, a large part remains unknown. According to our study findings, more adaptive and innate immune cells such as macrophages, monocytes, B cells, and immature dendritic cells infiltrated unstable plaques than stable plaques, which is in accordance with previous studies.^[61]^ In AS, white blood cell recruitment occurs when endothelial cells are stimulated by various triggers. The continuous migration of monocytes into the vascular subendothelial area results in their development into macrophages, which gobble up oxidized low-density lipoproteins and turn them into lipid-rich foam cells that survive within plaques and encourage the growth of AS. Macrophages’ ability to remove lipoproteins is definitely beneficial at the onset of AS. However, owing to the absence of negative feedback from consumption, these macrophages become lipid-engorged and form a sizable necrotic lipid core, which causes plaque destabilization. Lipid metabolic imbalances can jeopardize important immune activities.^[10,62]^

It was first reported in 2004 that NET formation is a neutrophil defense mechanism by killing pathogens.^[16]^ Since then, several studies have focused on NETs development. Besides entrapping and eliminating a wide range of microorganisms that cause infectious diseases, there is increasing evidence that NETs are also involved in various noninfectious diseases, including AS,^[63]^ thrombosis,^[64]^ autoimmune diseases,^[65]^ cancer,^[66]^ and diabetes mellitus.^[67]^ NETs are essential for promoting the development of atherosclerotic lesions and plaque instability.^[68,69]^ Atheromatous lesion size is reduced and the time for carotid artery thrombosis is postponed in ApoE^−/−^ mice when NET synthesis is inhibited by the PAD4 inhibitor chloro-amidine^[28,29]^ or when NET degradation is promoted by deoxyribonuclease I treatment.^[54]^ In our study, we identified 8 DE-NRGs by comparing 327 DEGs between unstable and stable plaques and 235 NRGs, all of which were significantly upregulated in vulnerable plaques. According to GO and KEGG analyses, these genes were predominantly engaged in the complement receptor-mediated signaling pathway, vascular endothelial growth factor signaling pathway, Fc epsilon RI signal transduction pathway, and leukocyte transendothelial migration. Studies have shown that complement factor C5a receptor activation causes neutrophils to generate NETs.^[70]^ Moreover, studies on pro-atherosclerotic action of IgE antibodies secreted by B cells mainly come from research on the Fc ε receptor. Wang et al^[71]^ found that in ApoE^−/−^ mice with Fc ε R1-α knockout, the atherosclerotic area of the aorta and the components of macrophages, T cells, apoptotic cell remains, and pro-inflammatory factor interleukin (IL)-6 in plaques were significantly decreased. Most peripheral white blood cells in the human immune system, neutrophils, are the first to reach the site of inflammation, where they exert pro-atherogenic effects by releasing NETs.^[72]^ Moreover, NETs can activate dendritic cells (DCs), encourage Th1 polarization,^[73]^ and enhance macrophage infiltration in patients or mice with diabetes.^[74]^ This is consistent with our observation that samples from unstable plaques had increased levels of immune cells such as macrophages, monocytes, DCs, B cells, and Th1 cells. These findings indicate an increased immune inflammatory process and NET generation in unstable atheromatous plaques compared to stable plaques.

Megens et al^[75]^ first confirmed and reported the presence of NETs in atherosclerotic lesions in mice and humans. In recent years, NETs have been confirmed to exist in the thrombosis of the involved vessels in patients with acute myocardial infarction with ST-segment elevation, and the degradation of NETs by deoxyribonuclease can reduce the atherosclerotic plaque.^[75,76]^ Franck et al^[25]^ conducted an in-depth analysis of 56 categories of human atherosclerotic plaques, and the results of their study showed that NETs were widely distributed in areas rich in apoptotic endothelial cells and SMCs. As for the mechanism of NETs on plaque instability, histone H4 contained in NETs can mediate SMCs membrane cleavage and lead to plaque instability. On the contrary, neutralizing histone H4 can prevent SMCs death and stabilize the plaque.^[69]^ Infiltration of immune cells, such as macrophages, Th cells, and B lymphocytes, is a major factor in the development of AS and plaque rupture. Studies have found that neutrophils induce endothelial cells to express monocyte chemoattractant protein-1 and intercellular adhesion molecule-1 by releasing NETs, which aggregate macrophages and promote atherosclerotic lesions.^[77]^ Megens et al^[75,78]^ found that NETs activate the autoimmunity of plasmacytoid dendritic cells (pDCs) in atherosclerotic lesions, cause a large amount of IFN-I synthesis and release, and then strengthen the inflammatory response to promote the progression of AS. In contrast, the same proatherogenic effect was not observed in mice treated with the PLX3397 antibody (which deleted neutrophils in the animals).^[68]^ Warnatsch et al^[54]^ demonstrated that cholesterol crystals trigger the release of NETs from neutrophils, while NETs induce the release of cytokines from macrophages and activate Th17 cells to enhance the aggregation of immune cells in atherosclerotic plaques. These studies have confirmed that NETs can promote the progression and instability of atherosclerotic plaques by activating and enhancing the aggregation of immune cells.

We also investigated the expression profiles of stabilized and destabilized atherosclerotic plaques. Additional investigations found that inflammatory genes such as CCL19 and MMP9 were elevated, whereas vascular smooth muscle cell contraction-associated genes such as MYH10 and MYH11 were reduced in vulnerable plaques. Functional enrichment analysis revealed that the immune response and extracellular matrix (ECM) may be linked to plaque vulnerability, indicating that SMCs and ECM, in addition to immune cells, have an impact on plaque destabilization. Under normal circumstances, SMCs are believed to maintain atherosclerotic plaque stability by producing and depositing ECM proteins. Nevertheless, a chronic inflammatory environment causes phenotypic transformation of SMCs, and synthetic SMCs secrete various MMPs capable of degrading the ECM.^[79,80]^ Based on DE-NRGs, differential expression analysis found significant differences between unstable and stable plaques. We sought to predict the stability of plaques based on DE-NRGs to distinguish individuals with AS who are at an increased risk of acute cardiovascular events for early prevention and therapy. By combining 3 machine learning approaches, we identified 4 characteristic genes associated with plaque destabilization, including C5AR1, FPR3, AQP9, and SIGLEC9. Some of these genes contribute to atherosclerotic plaque vulnerability based on NET formation. For example, the archetypal G protein-coupled receptor C5aR1 is encoded by C5AR1 and interacts with C5a, one of the most powerful inflammatory proteins in the complement system. According to research by Nathalie Niyonzima et al,^[81]^ atherosclerotic plaques have higher C5aR1 levels than normal arteries. Therefore, when carotid plaques were primed with C5a before being exposed to cholesterol crystals, they released more IL-1α, IL-1β, and IL-18. These results revealed that C5aR1 may accelerate AS development by triggering the nucleotide-binding domain and leucine-rich repeat protein 3 inflammasome. Neutrophil elastase (NE), a component of NETs, has C5 convertase activity that cleaves C5 proteins, thereby generating C5a, which in turn induces NET formation and results in atherosclerotic plaque instability.^[82]^ According to previous studies, AQP9 is mostly present in normal hepatic cells and neutrophils, where it controls lipid absorption, energy metabolism, cell migration, and inflammatory processes.^[83]^ In accordance with the findings of our investigation, Inouye et al^[84]^ discovered that AQP9 expression in the hepatic tissue of mice with atherosclerotic lesions was associated with the size of these lesions and was upregulated in human arterial tissue and atherosclerotic plaques. Formyl peptide receptors (FPRs) belong to the G protein-coupled receptor subfamily and are predominantly expressed in leukocytes.^[85]^ Three FPRs, FPR1-FPR3, in humans and their putative corresponding counterparts in mice have been identified.^[86]^ FPR1 and FPR2 are expressed on the surfaces of white blood cells and many non-hematopoietic cells and participate in multiple biological processes, such as inflammation control, tissue repair, and angiogenesis.^[86]^ However, while FPR3 is expressed in monocytes and DCs, its overall function remains unclear. Siglecs are a series of sialic acid-binding immunoglobulin-like receptors.^[87]^ The human SIGLEC9 mouse ortholog Siglec-E is primarily expressed in myeloid cells, such as neutrophils, macrophages, and dendritic cells.^[88]^ Hsu et al^[89]^ showed that the knockout of the Siglec-E gene accelerates AS in ApoE^−/−^ mice. Moreover, Siglec-E deficiency enhances the absorption of normal and oxidized lipoproteins, thereby promoting foam cell generation in vitro. These data suggested that Siglec-E has a preventive function against the onset and progression of AS. This finding seems inconsistent with our results. However, it is precisely because SIGLEC9 plays a protective role in the onset and progression of AS that it is up-regulated in atherosclerotic plaques that have already been formed for protection.

Unfortunately, this study had some limitations. First, despite the thoroughness of the bioinformatics analysis employed in this investigation, the conclusions require cautious evaluation because they lack experimental validation and randomized clinical trial data. Second, the establishment of NET-related gene sets requires further development as NETs research continues to advance. Finally, we conducted a transcriptome-based investigation of immune cell infiltration. Therefore, we were unable to determine whether the immune cells were the result of NET generation or if they were engaged in the process of NET formation. Additional studies are required to elucidate these fundamental mechanisms.

## 5. Conclusion

We identified several promising atherosclerotic plaque vulnerability-related NRGs (AQP9, C5AR1, FPR3, and SIGLEC9), and infiltrating immune cell subtypes, which may be capable of identifying vulnerable atherosclerotic plaques at an early stage and preventing various complications of plaque disruption.

## Acknowledgments

The open-access databases used in this research were provided by GEO, and we would like to appreciate them for that. We would like to thank Editage (www.editage.cn) for English language editing.

## Author contributions

**Conceptualization:** Tingting Hu, Xiaomin Chen.

**Data curation:** Tingting Hu.

**Formal analysis:** Tingting Hu.

**Investigation:** Tingting Hu.

**Methodology:** Tingting Hu.

**Software:** Tingting Hu.

**Validation:** Tingting Hu, Xiaomin Chen.

**Visualization:** Tingting Hu.

**Writing – original draft:** Tingting Hu.

**Funding acquisition:** Xiaomin Chen.

**Project administration:** Xiaomin Chen.

**Supervision:** Xiaomin Chen.

**Writing – review & editing:** Xiaomin Chen.

## Supplementary Material












