# Supplementary material for: Role of neutrophil extracellular trap and immune infiltration in atherosclerotic plaque instability: Novel insight from bioinformatics analysis and machine learning
Source: Medicine (Baltimore). 2023 Sep 22;102(38):e34918. doi: 10.1097/MD.0000000000034918 (PMC10519497; doi:10.1097/MD.0000000000034918)
Supplement: Supplementary file 6 [file medi-102-e34918-s006.pdf]

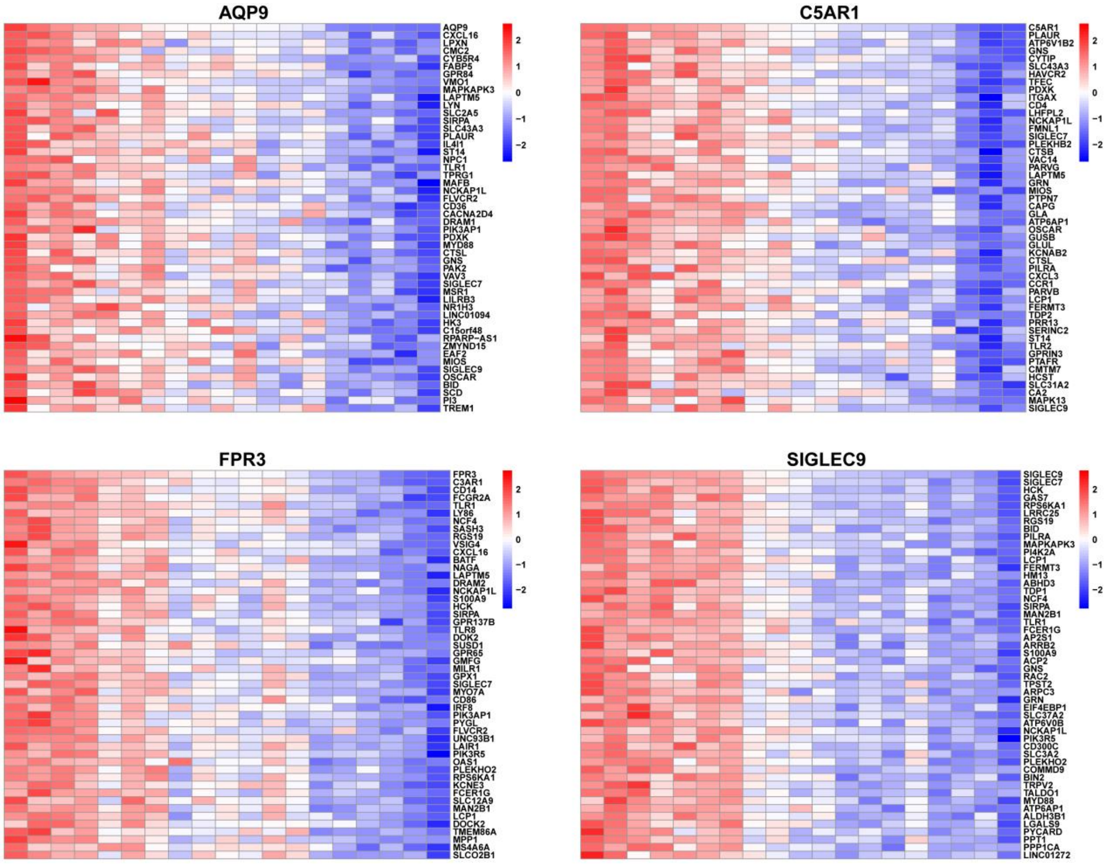

Supplemental Figure S1

Correlation analysis between characteristic genes and all genes involved in atherosclerotic plaques. Heatmaps display the top 50 atherosclerotic plaque-related genes positively associating with characteristic genes, respectively. Each row indicates distinct atherosclerotic plaque-related genes, and each column represents one of atherosclerotic plaque specimens.
